# Supplementary material for: Assessment of breast cancer surgical margins with multimodal optical microscopy: A feasibility clinical study
Source: PLoS One. 2021 Feb 11;16(2):e0245334. doi: 10.1371/journal.pone.0245334 (PMC7877783; doi:10.1371/journal.pone.0245334)
Supplement: Attachment — Submitted filename: PONE-D-20-28159.pdf [file pone.0245334.s001.pdf]

PLOS ONE

Assessment of breast cancer surgical margins with multimodal optical microscopy: a preliminary clinical study

--Manuscript Draft--

Manuscript Number:	PONE-D-20-28159
Article Type:	Research Article
Full Title:	Assessment of breast cancer surgical margins with multimodal optical microscopy: a preliminary clinical study
Short Title:	Optical microscopy for breast cancer surgical margins
Corresponding Author:	Nicusor Iftimia, PhD<br>Physical Sciences Inc<br>N Chelmsford, MA UNITED STATES
Keywords:	Breast cancer, surgical margins analysis, optical microscopy
Abstract:	Providing surgical margins information during breast cancer surgery is crucial for the success of the procedure. The margin is defined as the distance from the tumor to the cut surface of the resection specimen. The consensus among surgeons and radiation oncologists is that there should be no tumor left within 1-2 mm from the surface of the surgical specimen. If a positive margin remains, there is substantial risk for tumor recurrence, which may also result in potentially reduced cosmesis and eventual need for mastectomy.<br>In this paper we report a novel multimodal optical imaging instrument based on combined high-resolution confocal microscopy-optical coherence tomography imaging for assessing the presence of positive margins on surgical specimens. Since rapid specimen analysis is critical during surgery, we also assessed the use of a custom-made contrast agent, which enables enhanced more rapid identification of the areas of the specimen that have potential positive margins. Thus, we limit the use of high-resolution optical imaging to only those areas for visualizing tissue morphology at the sub-cellular scale and confirming or ruling out cancer presence. Preliminary evaluation of this technology was performed on 14 surgical specimens and testing of optical imaging findings was performed against histopathology. The combination of the three imaging modes allowed for high correlation between optical image analysis and histological ground-truth.
Order of Authors:	Nicusor Iftimia, PhD
	Savitri Krishnamurthy
	Gopi Maguluri
	Dorin Preda
	Jesung Park
	John Grimble
	Min Song
	Kechen Ban
	Mark Scimone
Opposed Reviewers:	
Additional Information:	
Question	Response
**Financial Disclosure**<br><br>Enter a financial disclosure statement that describes the sources of funding for the	Nicusor Iftimia-PI<br>2R44CA173998-02A1<br>US National Cancer Institute<br>The funders had no role in study design, data collection and analysis, decision to

Powered by Editorial Manager® and ProduXion Manager® from Aries Systems Corporation

work included in this submission. Review the submission guidelines for detailed requirements. View published research articles from *PLOS ONE* for specific examples.<br><br>This statement is required for submission and **will appear in the published article** if the submission is accepted. Please make sure it is accurate.<br><br>**Unfunded studies**<br>Enter: *The author(s) received no specific funding for this work.*<br><br>**Funded studies**<br>Enter a statement with the following details:<br>• Initials of the authors who received each award<br>• Grant numbers awarded to each author<br>• The full name of each funder<br>• URL of each funder website<br>• Did the sponsors or funders play any role in the study design, data collection and analysis, decision to publish, or preparation of the manuscript?<br>• **NO** - Include this sentence at the end of your statement: *The funders had no role in study design, data collection and analysis, decision to publish, or preparation of the manuscript.*<br>• **YES** - Specify the role(s) played.<br><br>* typeset	publish, or preparation of the manuscript.
**Competing Interests**<br><br>Use the instructions below to enter a competing interest statement for this submission. On behalf of all authors, disclose any competing interests that could be perceived to bias this work—acknowledging all financial support and any other relevant financial or non-financial competing interests.<br><br>This statement **will appear in the published article** if the submission is accepted. Please make sure it is accurate. View published research articles from *PLOS ONE* for specific examples.	The authors have declared that no competing interests exist.

**NO authors have competing interests**<br><br>Enter: *The authors have declared that no competing interests exist.*<br><br>**Authors with competing interests**<br><br>Enter competing interest details beginning with this statement:<br><br>*I have read the journal's policy and the authors of this manuscript have the following competing interests: [insert competing interests here]*<br><br>* typeset	
**Ethics Statement**<br><br>Enter an ethics statement for this submission. This statement is required if the study involved:<br><br>• Human participants<br>• Human specimens or tissue<br>• Vertebrate animals or cephalopods<br>• Vertebrate embryos or tissues<br>• Field research<br><br>Write "N/A" if the submission does not require an ethics statement.<br><br>General guidance is provided below. Consult the submission guidelines for detailed instructions. **Make sure that all information entered here is included in the Methods section of the manuscript.**	MD Anderson Cancer Center IRB Committee<br>IRB PA-14-1036 MDACC

Powered by Editorial Manager® and ProduXion Manager® from Aries Systems Corporation

**Format for specific study types**<br><br>**Human Subject Research (involving human participants and/or tissue)**<br>• Give the name of the institutional review board or ethics committee that approved the study<br>• Include the approval number and/or a statement indicating approval of this research<br>• Indicate the form of consent obtained (written/oral) or the reason that consent was not obtained (e.g. the data were analyzed anonymously)<br><br>**Animal Research (involving vertebrate animals, embryos or tissues)**<br>• Provide the name of the Institutional Animal Care and Use Committee (IACUC) or other relevant ethics board that reviewed the study protocol, and indicate whether they approved this research or granted a formal waiver of ethical approval<br>• Include an approval number if one was obtained<br>• If the study involved *non-human primates*, add *additional details* about animal welfare and steps taken to ameliorate suffering<br>• If anesthesia, euthanasia, or any kind of animal sacrifice is part of the study, include briefly which substances and/or methods were applied<br><br>**Field Research**<br><br>Include the following details if this study involves the collection of plant, animal, or other materials from a natural setting:<br>• Field permit number<br>• Name of the institution or relevant body that granted permission	
**Data Availability**<br><br>Authors are required to make all data underlying the findings described fully available, without restriction, and from the time of publication. PLOS allows rare exceptions to address legal and ethical concerns. See the PLOS Data Policy and FAQ for detailed information.	Yes - all data are fully available without restriction

A Data Availability Statement describing where the data can be found is required at submission. Your answers to this question constitute the Data Availability Statement and **will be published in the article**, if accepted.<br><br>**Important:** Stating 'data available on request from the author' is not sufficient. If your data are only available upon request, select 'No' for the first question and explain your exceptional situation in the text box.<br><br>Do the authors confirm that all data underlying the findings described in their manuscript are fully available without restriction?	
**Describe where the data may be found in full sentences. If you are copying our sample text, replace any instances of XXX with the appropriate details.**<br><br>• If the data are **held or will be held in a public repository**, include URLs, accession numbers or DOIs. If this information will only be available after acceptance, indicate this by ticking the box below. For example: *All XXX files are available from the XXX database (accession number(s) XXX, XXX.*).<br>• If the data are all contained **within the manuscript and/or Supporting Information files**, enter the following: *All relevant data are within the manuscript and its Supporting Information files.*<br>• If neither of these applies but you are able to provide **details of access elsewhere**, with or without limitations, please do so. For example:<br><br>*Data cannot be shared publicly because of [XXX]. Data are available from the XXX Institutional Data Access / Ethics Committee (contact via XXX) for researchers who meet the criteria for access to confidential data.*<br><br>*The data underlying the results presented in the study are available from (include the name of the third party*	All relevant data are within the manuscript

and contact information or URL).<br>• This text is appropriate if the data are owned by a third party and authors do not have permission to share the data.<br><br>* typeset	
Additional data availability information:	

Dear Editor,

We are very pleased to submit a Plos One paper manuscript entitled " *Assessment of breast cancer surgical margins with multimodal optical microscopy: A preliminary clinical study*".

This paper presents the results of a preliminary research study regarding the automated identification of positive margins on surgical specimens, which we believe will be of high interest for the biomedical research community. We will appreciate a timely feedback from reviewers.

Thank you for considering this manuscript.

Sincerely,

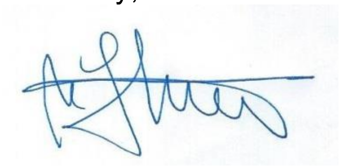

Dr. Nicusor Iftimia, Senior OSA Felow

Manuscript Click here to access/download;Manuscript;Plos One Sept 06 2020.docx

Assessment of breast cancer surgical margins with multimodal optical microscopy: A preliminary clinical study

MARK T. SCIMONE,[1] SAVITRI KRISHNAMURTHY[2], GOPI MAGULURI,[1] DORIN PREDA[1], JESUNG PARK,[1] JOHN GRIMBLE,[1] MIN SONG[1], KECHEN BAN, AND NICUSOR IFTIMIA[1*]

[1]*Physical Sciences Inc, 20 New England Business Center Drive, Andover, MA -01810*
[2] *University of Texas MD Anderson Cancer Center, Department of Pathology, 1515 Holcombe Blvd, Houston, TX 77030*
**iftimia@psicorp.com*

Abstract: Providing surgical margins information during breast cancer surgery is crucial for the success of the procedure. The margin is defined as the distance from the tumor to the cut surface of the resection specimen. The consensus among surgeons and radiation oncologists is that there should be no tumor left within 1-2 mm from the surface of the surgical specimen. If a positive margin remains, there is substantial risk for tumor recurrence, which may also result in potentially reduced cosmesis and eventual need for mastectomy.
In this paper we report a novel multimodal optical imaging instrument based on combined high-resolution confocal microscopy-optical coherence tomography imaging for assessing the presence of positive margins on surgical specimens. Since rapid specimen analysis is critical during surgery, we also assessed the use of a custom-made contrast agent, which enables enhanced more rapid identification of the areas of the specimen that have potential positive margins. Thus, we limit the use of high-resolution optical imaging to only those areas for visualizing tissue morphology at the sub-cellular scale and confirming or ruling out cancer presence. Preliminary evaluation of this technology was performed on 14 surgical specimens and testing of optical imaging findings was performed against histopathology. The combination of the three imaging modes allowed for high correlation between optical image analysis and histological ground-truth.

1. Introduction

In breast cancer cases that opt for breast-conserving surgery, it is extremely important to achieve negative margins around the primary tumor. The margin is defined as the distance from the tumor to the cut surface of the resection specimen. The consensus among surgeons and radiation oncologists is that there should be no tumor left within 2 mm from the surface of the surgical specimen **(1–3)** in patients with ductal carcinoma in situ and no tumor at the inked margin in patients with invasive mammary carcinoma. If a positive margin remains, there is substantial risk for tumor recurrence **(4,5)**. Published reports indicate a 20-70% rate of positive margins left after lumpectomy **(6–8)**. The required repeated surgery in these cases may result in potentially reduced cosmesis and eventual need for mastectomy if cancer recurrence is not rapidly detected.

Current techniques for intraoperative pathologic assessment involve touch prep and frozen section analysis **(9,10)**. Touch-prep or imprint cytology allows for cytologic evaluation of the whole lumpectomy surface and has good sensitivity and specificity **(11)**. However, this technique is time consuming, is cumbersome, requires special expertise, and does not detect tumor foci close to the lumpectomy surface (residual cells < 2mm from the margin). Frozen section analysis is a technically challenging procedure due to the significant amount of fatty tissue found in breast specimens, which makes it difficult to freeze the tissue effectively. While the best results using this technique have been shown to reduce the rate of second operations to about 20% **(12)**, false negatives still occur at high frequencies **(13)**. Due to these

difficulties, the vast majority of hospitals in the U.S. do not perform intra-operative margin assessment .

Clinical research approaches for intraoperative margin assessment of breast specimens include micro-CT, MRI, high frequency ultrasound, positron emission tomography (PET), and radiofrequency spectrometry. MRI and micro CT do not provide a cellular-level picture of the tissue microstructure that is necessary to assess margins accurately and therefore their sensitivity is not very high **(14–16)**. Ultrasound guidance for surgical margins can be useful for determining margin status in a low-cost manner, especially for women with dense breasts where other methods may not work as well. However, its reported % sensitivity is rather modest (~44% in some studies) **(17)**. PET has shown a sensitivity of 92% to 96% and a specificity of 84% to 91% for breast margins assessment **(18,19)**. However, PET is expensive, time-consuming, and invasive, which makes it not appealing for intraoperative margin assessment of breast surgical specimens. Radio-frequency spectrometry, has had limited sensitivity (60% or less) for positive margins and can only investigate relatively small surface areas (~2 mm) **(20)**.

Perhaps discuss the discrepancy between the poor sensitivity and the reduced reoperation rate with this tech?

Optical imaging techniques have been investigated for evaluation of physiological, chemical, and morphological changes associated with cancer so as to facilitate margin assessment. These techniques include fluorescence imaging, Raman spectroscopy, optical coherence tomography (OCT), and photoacoustic tomography, among others **(21–28)**. Although most of these techniques achieved high accuracy in cancer delineation, they have not yet been translated into routine clinical practice due to some inherent pitfalls, such as reduced sensitivity, limited speed, inability to quickly cover a large tissue area, and requirement of expert operator skills **(29)**.

However, it has been noted that some of the optical modalities provide complementary information, and thus their combined use might help to improve sensitivity, and even speed for evaluation of large surface areas of surgical resection specimens. Therefore, this paper aims to introduce a novel multimodal microscopy device that might enable more accurate detection of breast tumor margin at a reasonable speed. By combining fluorescent microscopy (FM), optical coherence tomography (OCT), and reflectance confocal microscopy (RCM), the platform provides rapid delineation and high-resolution assessment of tumor margins with minimal feedback from operator. Briefly, using a fluorescent cancer targeting agent, FM highlights regions-of-interest (ROIs) of likely positive margins. While providing a large field of view and relatively rapid analysis of the specimens, the specificity of FM is not very high (usually prone to false positives). Therefore, combined OCT/RCM imaging is used as well to analyze the ROI at submicron scale resolution to confirm or rule out cancer presence. RCM is used to determine if the fluorescence imaging highlighted margins are positive, while OCT is used to rapidly determine their extent in the lateral and deep aspects. In this way, the system is optimized for rapid acquisition time, keeping intraoperative utility in mind as a primary objective. The multimodal instrument and software for image analysis developed by us were evaluated on breast tissues obtained from surgical resection specimens encountered in clinical practice. The detected margins were compared with the ground truth of histopathological examination of the imaged tissue. Fourteen total specimens were analyzed for this initial report. Strong correlation with histology (100%) was found in all specimens that presented positive cancer margins (6 of the 14 total). The imaging of the benign specimens also correlated well with the histology. However, OCT was prone to false positives in a small number of specimens, particularly in high scattering areas of normal lobules. Further careful inspection of the specimen with high resolution RCM allowed for accurate classification of the tissue.

2. Materials and methods

2.1 Fluorescence imaging agent. A highly sensitive contrast agent (see structure in Fig. 1) consisting of an optically silent peptide substrate containing two (near-infrared; NIR)

fluorochromes (Cy5.5), internally quenched, was synthesized to track positive margins on breast lumpectomy specimens via specific uPA cleavage.

define

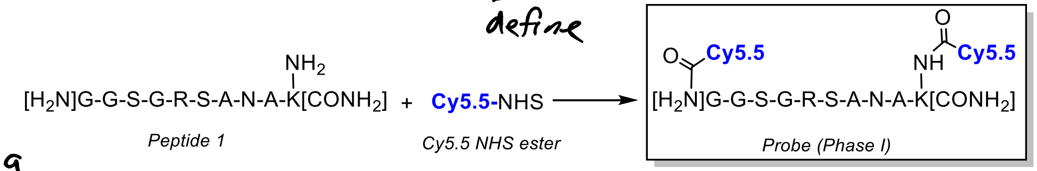

Figure 1: Contrast Agent synthesis and structure.

was inflammation a problem with uPA on its own? Did this help or why was increased specificity needed?

To further increase specific targeting of cancer tissue, a peptide moiety targeting the Her2 cancer receptors was appended (see Fig. 2). A spacer (aminohexanoic acid) was used to ensure that this peptide moiety will maintain fast uPA cleavage kinetics. The multifunctional peptide can be cleaved by highly expressed breast cancer enzymes, like uPA, to provide a fluorescence signal only ~~on the cancer areas~~. Once the quenched NIR agent is cleaved, the released mono-NIR dye species become fluorescent when activated by near-infrared light. Thus, after incubation, the fluorescence contrast ratio between the cancer and benign tissue is increased, as shown below.

in the regions of cancer.

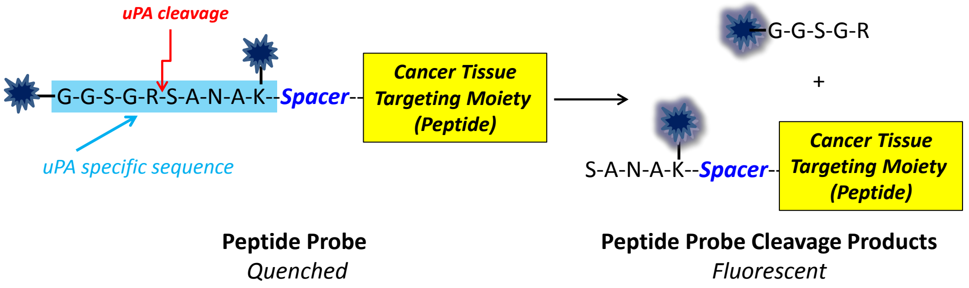

Figure 2: Peptide cleavage and fluorescence release

The targeting agent synthesized by our team showed 90% quenching efficiency and 10X fluorescence increase after cleavage (see Fig. 3). The activation time was tested as well using variable concentrations of the uPA enzyme. Based on literature data, we estimated the concentration of the enzyme that is present in malignant tissue versus benign tissue, and used these numbers as a baseline for our measurements. Literature reports (**30**) show that the average uPA level in benign tissue is 1 ng/mg protein, while in tumor tissue is 8 ng/mg protein. Considering an average volume of tissue of 5-10 cc, 1 mm penetration depth of the agent after incubation, 1 g/cc density (for breast tissue), and 1uM concentration of the contrast agent in saline solution, we determined that a ratio of 1:200 between the enzyme and substrate (E:S) is representative of benign tissue, while a ratio of 1:25 is representative of tumor tissue.

Check spacing

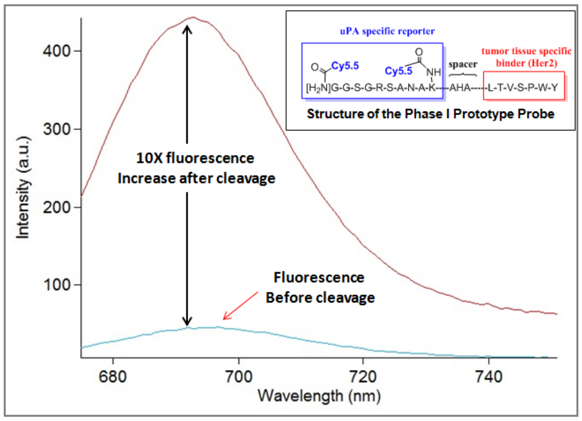

Fig. 3: Contrast agent structure and fluorescence

However, since the tumor tissue often infiltrates benign tissue, we also considered an intermediate ratio of 1:50. Using these ratios, we performed titration kinetic activation experiments to assess the fluorescence release as a function of time and E:S ratio. The results are outlined in Figure 4. As observed, the best contrast (4.5:1 for E:S=25 vs. E:S=200 and 3.5: for E:S =50 vs. E:S=200) was obtained after only 1 minute of incubation. After that, the cleavage begins at the lower (benign) concentration of uPA, resulting in approximately half the fluorescence ~~fold~~ increase as the malignant concentration. This suggests that that the optimum incubation time will be about 1 min, when the fluorescence ratio is over 3, providing sufficient contrast between the benign and tumor-benign mixed tissue. The unbound agent derived species are washed with saline after incubation to minimize any interference with the readout.

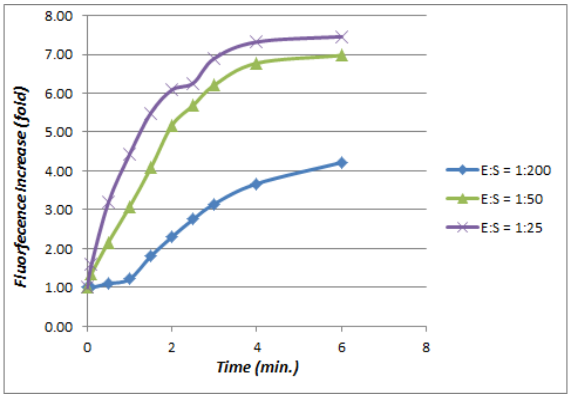

Figure 4: Contrast agent activation kinetics

2.2 Multimodal imaging system. A simplified schematic of the optical setup is shown in Fig.5 and photographs of the instrument are shown in Fig. 6.

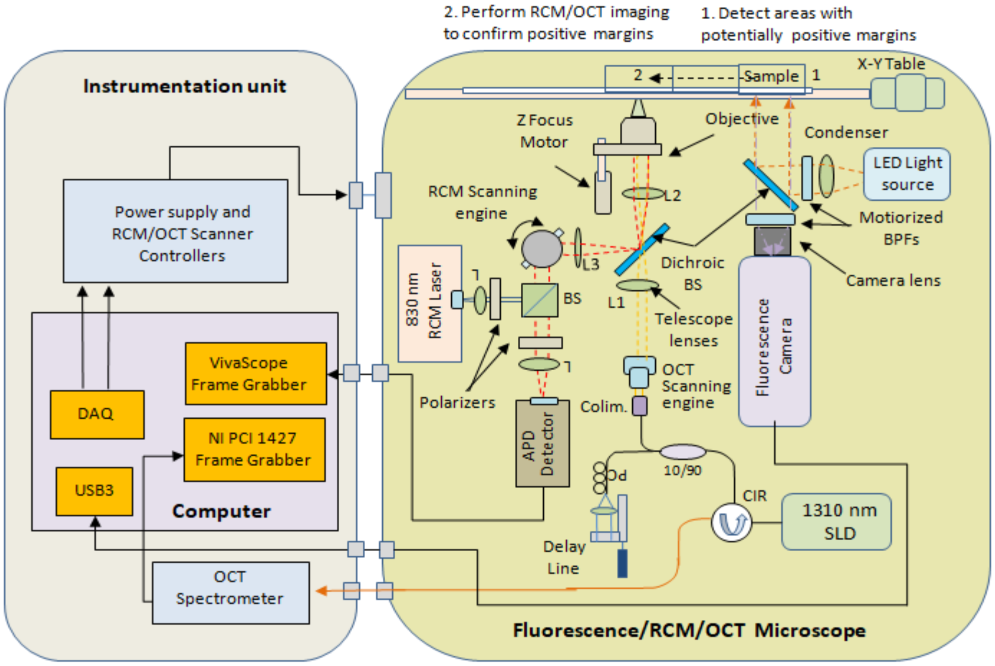

Figure 5. Simplified schematic of the multimodal optical imaging microscope

The FM subsystem consists of a high power LED Chip light source (Model Chanzon, 380nm-840nm / 3000mA), a highly sensitive NIR camera (Model PCO Edge 4.2, PCO Tech. Inc.), dichroic mirrors and band pass filters (Chroma Technologies) to properly match the excitation/emission bands of the CY 5.5 dye. The bandpass filter in front of the camera is

Is this large enough for the majority of expected specimens?

positioned on a motorized mount, such that a brightfield image can also be taken, and overlaid by the NIR image. The field of view (FOV) of the fluorescence channel is 1.5 x 1.5 inches. Following fluorescence ROI selection, the sample is automatically translated to be centered in the OCT/RCM objective field of view.

The RCM/OCT subsystem shares the same distal end optics (telescope lens and imaging objective), while in the upper stream the optical paths are separated by a dichroic mirror. Folding optics and telescope relay lenses are used in both the RCM and OCT optical paths such that the beam scanners can be relayed to the pupil plane of the imaging objective. The RCM subsystem is based on a typical confocal scheme. A resonant scanner (Model CR08, Cambridge Technlogies, MA) and a galvanometer scanner (Model 6200H, Cambridge Technologies, MA) are used to generate a raster scan on the sample surface.

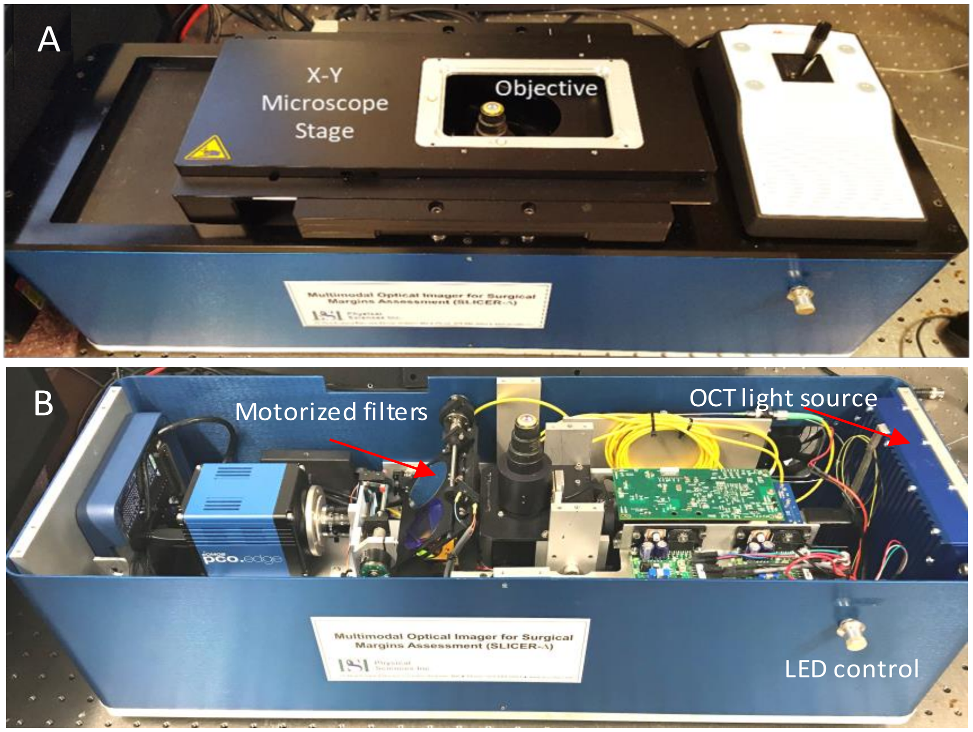

Figure 6. Photograph of the multimodal microscope. A. General view; B- Inside view

The two scanners are relayed to the imaging objective entrance pupil with two sets of relay lenses (telescope arrangement). The light from an 830 nm laser diode is directed to the scanners by a beam-splitter cube. The light returned from the sample is directed by the same beam-splitter cube to the avalanche photodiode (APD) detector. A pinhole is placed in front of the APD to reject the out-off-focus photons. Two polarizers are crossed in the emission and detecting paths to reduce the impact of sample specular reflections.

The OCT subsystem consists of a fiber optic (FO) interferometer with an optical delay line in the reference arm and a scanning engine in the sample arm. A superluminescent diode with 1310 nm central wavelength and approximately 100 nm 3dB bandwidth is used as the OCT light source. This provides a theoretical axial resolution of about 8 μm (in air), while the achieved resolution was 9 um due to imperfect balancing of the dispersion between the two arms of the interferometer. A fiber optic circulator is used to maximize light collection from the sample arm.

Both the OCT and the RCM signals are digitized and processed by a System Control and Data Processing unit. A specially-designed Vivascope frame grabber (Caliber ID, Rochester,

NY) is used to digitize and process the RCM signals, enabling a display rate of 9 frames/sec. A graphical processing unit (GPU), model GTX 1060 with 1152 CUDA cores is used to expedite the OCT data processing (FFT, dispersion, and interpolation), allowing for 40 frames/sec real-time display.

2.1 Software for instrument control, data acquisition, and processing

The instrument control software was written in LabVIEW, employing C-based dynamic link libraries for rapid processing and easy debugging. A user-friendly graphical interface allows for instrument setting, data acquisition and processing. The main steps for data collection and processing are illustrated in Fig. 7.

The bright field and subsequently the fluorescence images of the specimen are first collected (see Figs. 7A and 7B). Then, the user defines a region of interest (ROI) that incorporates the florescence image, as shown in Fig. 7B, and then the instrument automatically scans this area and collects spatially co-registered cross-sectional OCT images and enface RCM images (see Figs. 7D and 7C-C'). Since the size of the ROI is usually larger than the instantaneous field of view for OCT and RCM (2.2 mm for OCT and 0.75 mm for RCM), a line-strip imaging approach is used. The number of strips and their length is automatically calculated by the software as a function of the ROI size. An overlap between adjacent strips is user defined to enable the cross-correlation of the adjacent strips for full area image stitching.

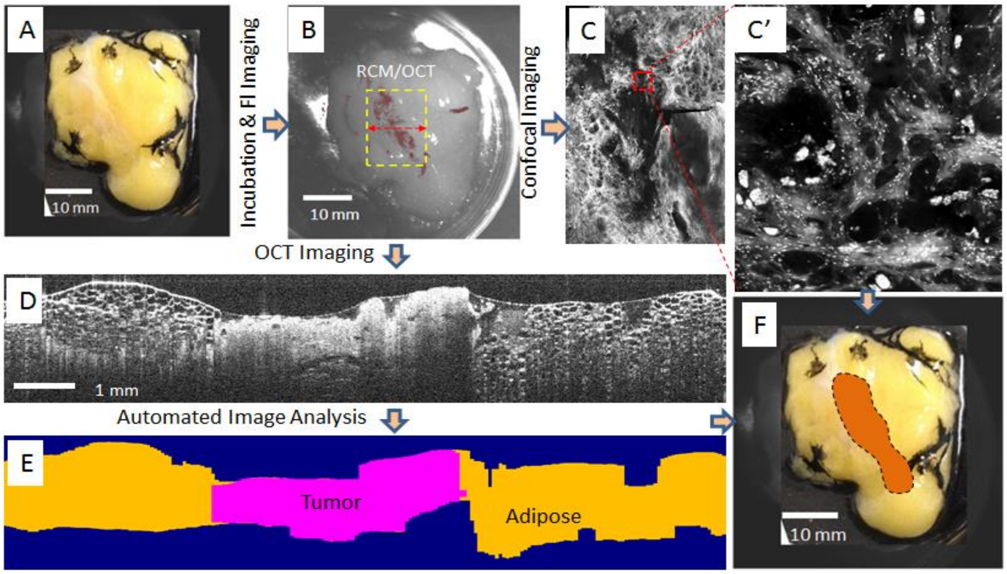

Fig 7. Main steps for data acquisition and processing. A: Bright field image of the specimen; B: False-color fluorescence imaging taken after specimen incubation with the contrast agent; C and C': RCM images and zoomed in region shown in red box, respectively. D: OCT image of one B-scan; E automated segmentation of the OCT data through full volumetric image (one cross section shown here); F- Overlay of the positive margins on the specimen picture.

Note that the RCM imaging over the entire area highlighted by FL imaging is not performed, as it will take too long time on large areas. Only small regions of interest (ROIs) are used to determine if areas indicated by fluorescence imaging are cancer positive or not. Once these areas are confirmed, OCT imaging and segmentation is performed to determine the extent of the positive margins. Tissue-type classification is based on the analysis of the RCM image. With minimal user feedback (assignment of distinctive tissue-types on 2-3 locations of the RCM image), an automated algorithm (see Section 2.2) analyzes the collected

images, determines tumor lateral and depth spreading on the OCT image (see cross-sectional tissue-type assignment on Fig. 7E), and overlays the tumor margins on specimen surface image (see Fig. 7F).

2.2 *Semi-automated software algorithm for tissue-type assignment*.

Following the collection and analysis of 3-4 RCM ROIs for each tissue type in different locations of the fluorescence highlighted area, such that both cancer and benign tissues are represented as determined by the pathologist, the overlapping enface OCT images are automatically assigned to the cancer and benign tissue types and used as training sets for the OCT image analysis algorithm. Representative training images are shown in Figure 8A.

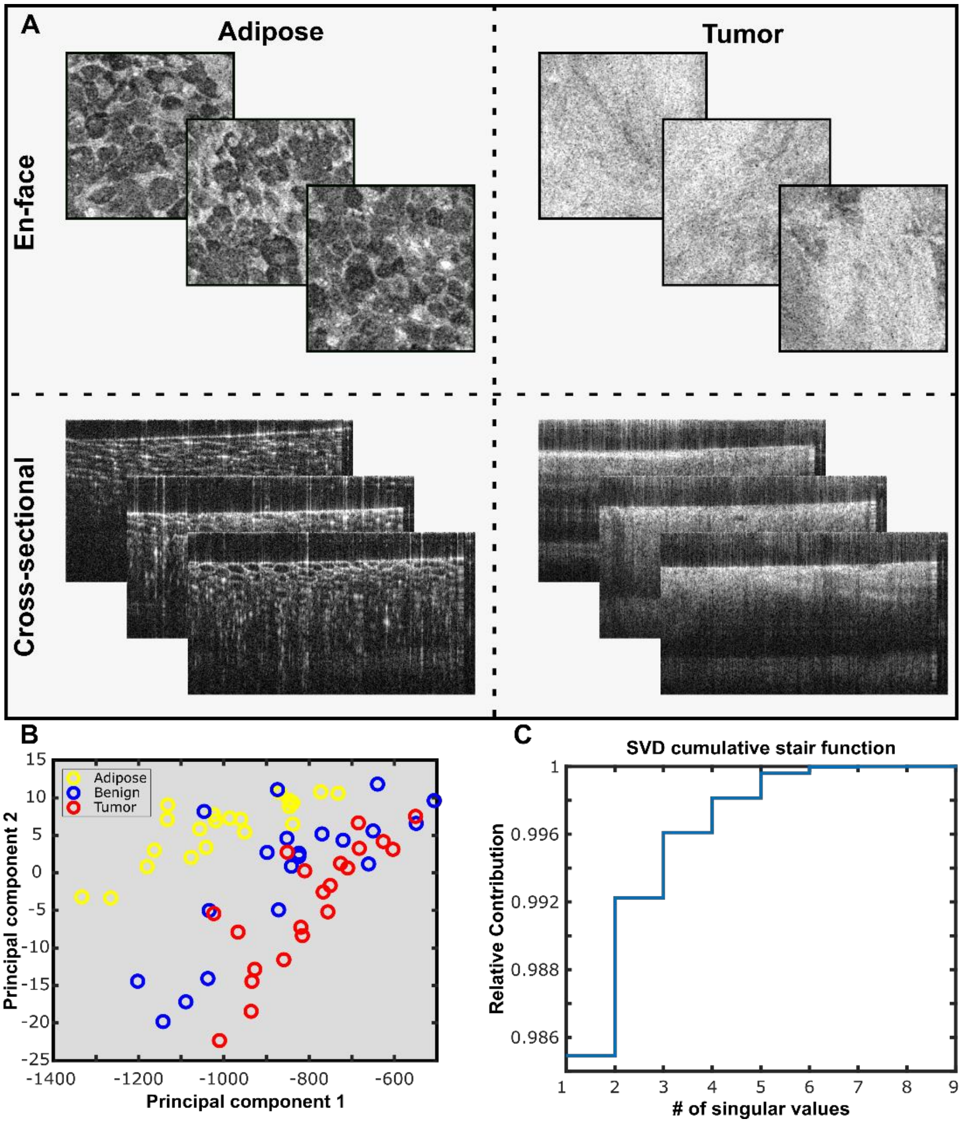

Fig 8: Simplified representation of the automated algorithm for tissue differentiation. **A.** Representative en-face and cross-sectional (b-scan) training images for adipose tissue and tumor tissue (benign representative images not shown for brevity). **B.** Principal component analysis of 9 spatial texture features shows some clustering in 2 strongest components, but

also considerable overlap. **C.** Singular value decomposition cumulative stair function showing that 5 or fewer parameters should enable strong classification capability.

Initial singular value decomposition and principal component analysis indicated that tissue slope and standard deviation of roughness are the most discriminating features.

The standard deviation of roughness and tissue slope were the most discriminating features, and their calculation is detailed further. Very high frequency information was smoothed using a Gaussian kernel. Then, to calculate the roughness, the waviness profile was first determined and subtracted from the filtered image. Finally, a standard deviation filter was convolved with the roughness image. Tissue type assignment based off standard deviation of roughness was determined using values from the training image sets. The tissue slope was computed through the Prewitt image gradient method. Again, tissue-type assignment based off tissue slope was determined using values from the training image sets. All analysis was done in MATLAB, where the analysis and plotting takes approximately 1 min. The processing duration can be further shortened by implementing this code in C++.

Three-dimensional tissue assignment (in cross-sectional images, and in different z-planes of en-face), required depth adjusted image processing. That is, signal drop in depth was calculated for each image so that tissue-type assignment thresholds could account for it. Additionally, in cross-sectional images, the top and bottom surfaces needed to be segmented. The top surface is highly reflective and thus can be segmented by a peak-finding algorithm. The bottom (or maximum signal depth) was segmented by a histogram-based region-property analysis.

2.3 *Tissue specimens collection and preparation*

Breast surgical specimens were provided by MD Anderson Cancer Center. Fourteen de-identified specimens were acquired from breast cancer patients undergoing surgery procedures (both lumpectomy and mastectomy) under the IRB approved protocol PA14-1036. Freshly collected specimens were incubated with the contrast agent for maximum of 2 minutes, washed with saline twice, and placed on the microscope tissue holder. The specimens were slightly pressed against the microscope window facing the imaging objective, to maximize the area of the specimen from which images were collected. This custom tissue holder is particularly important for RCM, as the depth penetration requires the specimen surface to be relatively flat and close to the objective.

3. Results

Of the 14 specimens analyzed, histology showed positive cancer margins on 6 specimens and negative margins in the remaining 8. Fluorescence imaging showed positive margins in 10 of the 14 specimens, so all specimens with positive margins were identified. This is very important as no positive margins were missed, as fluorescence was used to further guide RCM-OCT imaging. OCT imaging correctly identified all 6 specimens with positive margins and showed false positive margins on other two specimens (mainly on areas of normal lobule locations, which showed similar scattering and image patterns similar to the cancer areas). RCM imaging was used to test the OCT false-positives and it correctly identified the false positives, as it provided the resolution close to histopathology. We also noted that the algorithmic segmentation using OCT alone had correctly segmented other features, such as fibrosis.

Four representative examples of margins assessment on breast surgical specimens are shown in Figs. 9-12. The four cases were chosen to show distinct types of specimens that might be found during surgery: 1) specimens with positive margins all around, 2) heterogeneous specimens with infiltrating tumor margins, 3) negative margins specimens that visually contains only adipose tissue at its surface, 4) and heterogeneous benign specimens that after visual and tactile examination show areas similar to cancer.

The first case (see Fig. 9) is that of a fragment of a mastectomy specimen, which presented nearly complete positive margins, as it was not collected with the purpose of

preserving positive margins. Imaging was performed on a single surface, since visually the specimen showed similar tissue nature all around. The goal of imaging this specimen was to determine optical imaging/algorithm capability to correctly estimate cancer presence on the entire specimen surface, as indicated by histology. Indeed, as observed in the fluorescence image (Fig. 9B), nearly the entire specimen surface showed enhanced fluorescent signal relative to the background, suggesting uniform cancer presence at the specimen's surface. Cancer presence on over 80% of the specimen surface was confirmed by histology (see the red area annotated by the breast pathologist in Fig. 9C). The enface segmented OCT image taken near specimen surface (see Fig. 9D), as well the subsurface cancer presence, over a depth of approximately 1 mm, as shown projected on Fig. 9G, confirm a very similar cancer spreading area.

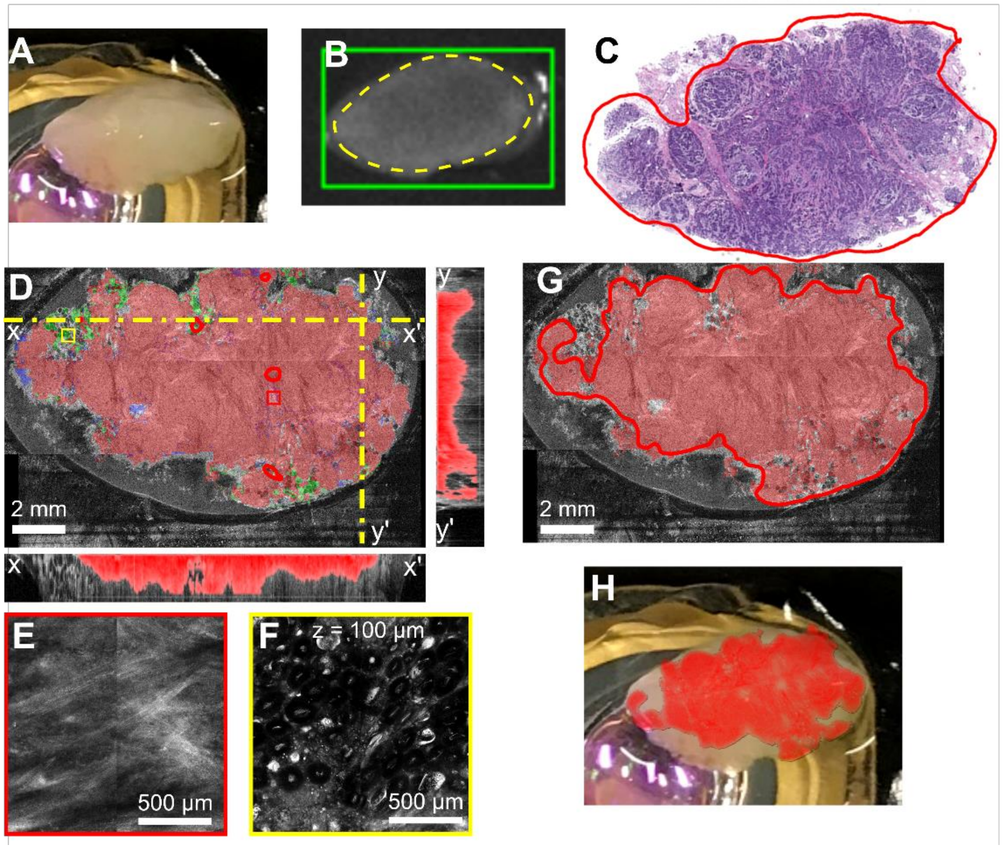

Fig. 9. Multimodal optical imaging findings on a small surgical resection specimen (~14 mm x 8mm). **A**. Camera image of the specimen on the microscope stage. **B**. Fluorescence imaging, where the green box shows the selected imaging ROI in the software, and the yellow-dotted line shows the area where there is higher fluorescence from the contrast agent. **C**. The histology slide, where red indicates invasive ductal carcinoma. **D**. Segmented enface OCT image showing cancer margins with corresponding X and Y cross-sectional images. Small red and yellow boxes show region of RCM images in panels E&F**.** Colored overlays shows algorithmic segmentation of tissues: tumor (red), adipose (green), and benign (blue). The cross-sectional images have segmented tumor overlaid in red. **G** Projection of the cancer areas from the multiple OCT slices over a depth of approximately 1 mm. **H.** Overlay of the positive margins on the surgical specimen.

The second case, shown in Fig. 10, is that of a small surgical specimen with positive margins (cancer infiltrating at the specimen's surface). A fluorescence signal was noticed on one of the faces of the tissue specimen, as shown in Fig. 10A. However, as confirmed by histology, the fluorescence area overestimated cancer presence, as the signal appears to be heightened in areas of adipose as indicated by the histopathology's-annotated areas in the histology slide (see Fig. 10C).

The segmented enface OCT image taken near specimen surface (see Fig. 10D), shows reasonable overlap with histology relative to cancer presence. Histology preparation led to loss of fatty areas towards the top of the specimen as the processing of the fatty tissue for histopathological sectioning is usually difficult. This is clear when comparing the histology to the OCT (Fig 10 D,G) and phone-camera image (Fig. 10 A). The cross sectional segmentation and Fig. 10G show that the area of cancer spreads as depth increases. This highlights the need for subsurface OCT imaging and volumetric segmentation. The RCM zoomed-in panels (Fig. 10E-F) show the structure in a tumor and fat area, respectively.

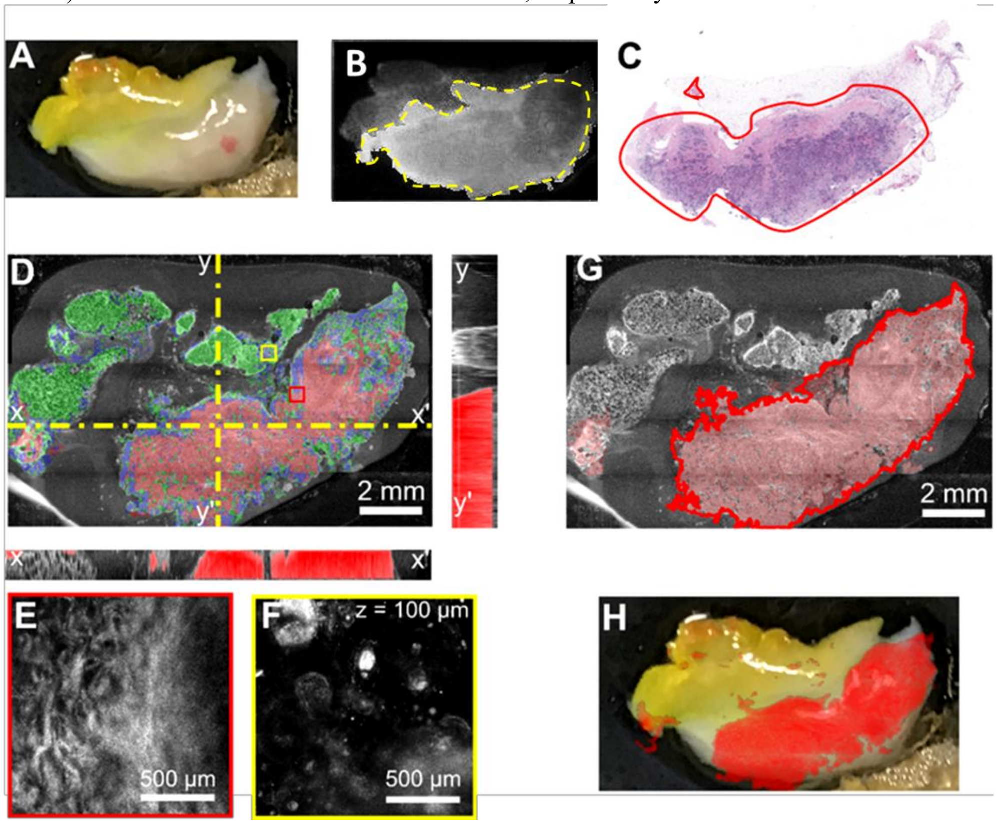

Fig 10. Example of positive margins identification on a small lumpectomy specimen. **A.** Camera image of the specimen on the microscope stage. **B.** Fluorescence imaging, where the red box shows the selected imaging ROI in the software, and the yellow-dotted line shows the area where there is higher fluorescence from the contrast agent. **C.** The histology slide, where red indicates invasive ductal carcinoma. **D.** Segmented enface OCT image with corresponding X and Y cross-sectional images. Dotted-yellow line shows the plane of the cross-sectional images. Colored overlays shows algorithmic segmentation of tissues: tumor (red), adipose (green), and benign (blue), Colored boxes show region of RCM images in panels **E-F**. The z-depth shown in **F** corresponds to the imaging depth for all RCM panels **G)** Projection of the cancer areas from the multiple OCT slices over a depth of approximately 1

mm shows larger predicted tumor areas at depth than in panel D. **H.** Overlay of the positive margins on the surgical specimen.

The third example is that of a surgical specimen free of positive margins (see Fig. 11), as indicated by the histology image (Fig. 11C), which is shown to have only a benign breast lobule (yellow) and normal duct (green). The fluorescence imaging, however, indicated a small area of potentially positive margins (see Fig. 11B, red asterisk).

Confocal imaging of the fluorescence-highlighted area has indicated the presence of a benign lobule. Although in the real clinical practice additional OCT imaging would be unnecessary when RCM indicates the absence of the positive margins, in this case we performed OCT imaging to confirm that the segmentation algorithm assigns this area to the correct tissue class. The enface OCT image (Fig. 11D) of the specimen surface clearly shows the presence of fat cells on over 90% of the specimen surface, as well as of a small area of a benign breast lobule (see blue area on the right upper area of Fig. 11D), as confirmed by RCM imaging (Fig. 11 E). Fig 11F confirms the presence of the fatty tissue on most of the specimen's surface, as indicated by the segmented OCT image (Fig. 11D).

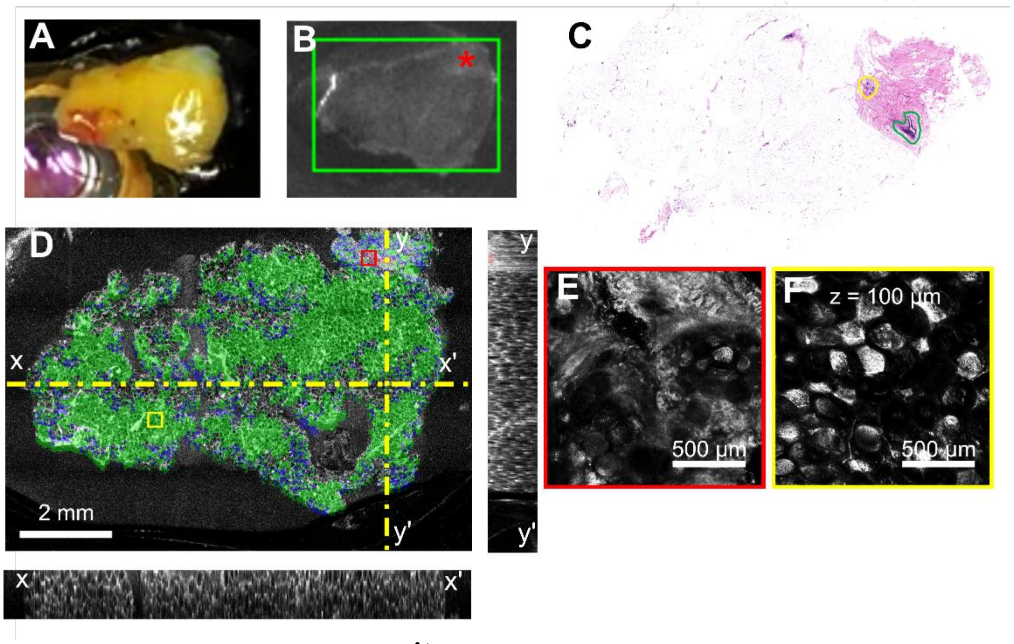

Fig 11. Example of negative margins identification on a small lumpectomy specimen. A) Camera photo of the specimen on the microscope stage. B) Fluorescence imaging seems to indicate positive margins presence (red asterisk). Green square indicates the imaging ROI selected in the software. C) Annotated histology indicating a benign breast lobule (yellow) and normal duct (green). D) Enface OCT image with corresponding X and Y cross-sectional images. Dotted-yellow line shows the plane of the cross-sectional images. Red and Yellow boxes show region of RCM images in E and F panels. Colored overlays shows algorithmic segmentation of tissues: tumor (red), adipose (green), and benign (blue). E) RCM image of lower part of the normal duct area. F) RCM image of adipose tissue. z-depth of RCM image is consistent at 100 μm.

The fourth case is that of a heterogeneous specimen, where multiple tissue types are apparent by eye (Fig 12A). After incubation with the fluorescent contrast agent, a small

region in the corner showed increased signal (Fig 12B), and thus warranted in depth analysis. The histological annotation shows the presence of multiple benign breast lobules (Fig 12C, blue circles). Again, these structural features contribute to small false positive tumor identification in the algorithmic segmentation. This is shown in Fig 12D&G. However, the RCM panel in Fig 12E was interpreted as benign by the ~~histologist~~ pathologist, showing how multiple imaging modes can inform a more accurate classification of the tissue.

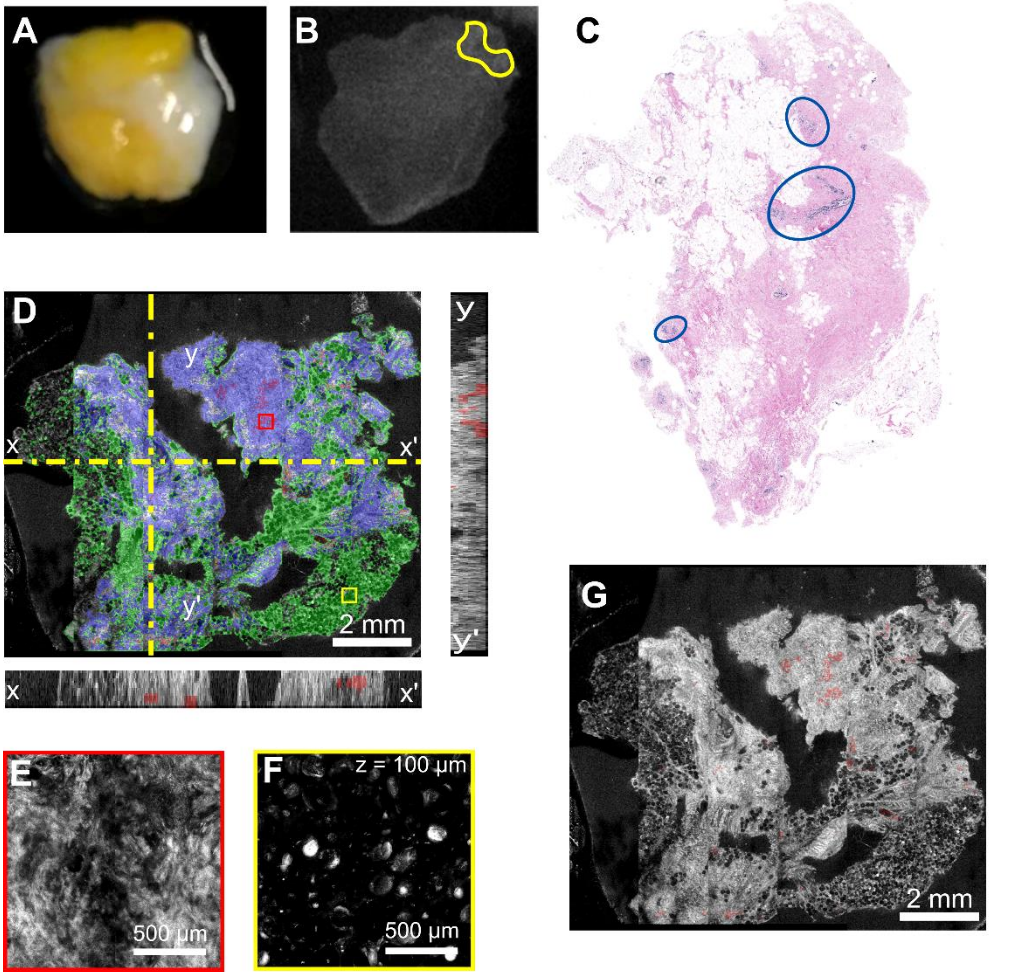

Fig 12. Example of negative margins identification and false positives on a heterogeneous benign specimen. **A)** Camera photo of the specimen on the microscope stage. **B)** Fluorescence imaging seems to indicate and area of tumor in the top-right corner (yellow). **C)** Annotated histology indicating benign breast lobules (blue). **D)** Enface OCT image with corresponding X and Y cross-sectional images. Dotted-yellow line shows the plane of the cross-sectional images. Red and Yellow arrowheads show the region of RCM images in e and f panels. Colored overlays shows algorithmic segmentation of tissues: tumor (red), adipose (green), and benign (blue). Colored boxes show region of RCM images in panels E & F. **E)** RCM image of lower part of the normal duct area. **F)** RCM image of adipose tissue. z-depth of RCM image is consistent at 100 µm. **G)** Enface OCT image with only the false positive algorithmic tumor predictions shown.

4. Discussion

Using our recently developed multimodal instrument, we examined breast surgical resections encountered in routine clinical practice for positive margins. Our novel instrument combines three optical imaging modalities (FL, RCM, and OCT) for margin assessment of breast specimens. Furthermore, a custom contrast agent was developed by attaching Cy5.5 to a quenched uPa vulnerable sequence. This was further attached to a cancer targeting moiety for effective staining of the tissue. Analysis of the cleaved increase in fluorescence shows strong increase in the presence of cancer in the tissue. The contrast agent and fluorescent microscopy gives an initial estimate of residual cancer on the surface of the tissue. However, because evaluation by fluorescence microscopy alone is limited only to surface information, and subject to minor non-specific signal, the utility of a combination of OCT and RCM becomes crucial in margin analysis. The RCM gives submicron detail to inform tissue-type classification at margins and interfaces, while the OCT provides volumetric mapping of the extent of the tumor infiltration. By using the FL mode to identify areas of highly suspicious for presence of cancer, the acquisition time can be greatly reduced during RCM/OCT imaging, which is critical for intraoperative utility. The information from the three modalities was combined in an image-processing regimen to automatically detect the cancer margins in 3D very effectively.

Four cases were highlighted in this paper to demonstrate the potential clinical utility of the multimodal optical imaging for breast cancer surgery guidance. The first case presented showed uniform staining with a near complete increase in FL signal from the contrast agent. RCM/OCT imaging and subsequent image analysis showed similar agreement, that the margins extended to the surface and edges and that the tumor content was distributed throughout the depth. Histopathological analysis confirmed that the specimen was an invasive ductal carcinoma, and the annotated section correlated well with the RCM/OCT images in space.

The second case was a more complicated specimen and more representative of the type of tissue found at tumor boundaries. Visual inspection showed a clear interface between yellow, likely fat and whiter, potentially malignant tissue. The FL image showed increased brightness from the contrast agent in approximately the same areas as observed visually, though the signal ratio was not as high as in case 1. The RCM/OCT imaging showed a more complicated tissue structure than was seen in case 1. The annotated histology of the hematoxylin and eosin stained tissue section indicated invasive ductal carcinoma amidst normal breast tissue. At certain depths, the histological annotations matched well with the image analysis prediction. However, through the analysis of the volumetric OCT data, we predict the tumor extended laterally in depth. In this study, the specimens were sectioned only a limited number of times. For 3D ground truth from histology, the specimen would need to be sectioned serially throughout, which is costly, introduces delays, and requires lengthy expert interpretation. This emphasizes that further improvements of this instrument is feasible which will be useful for eventual incorporation into surgical pathology practice.

The third presented case is one that visually appears like uniform adipose tissue. The FL channel did not show increased fluorescence signal and the histopathologist indicated no tumor content Although in the real clinical practice additional OCT imaging would be unnecessary when RCM indicates the absence of the positive margins, in this case we performed OCT imaging to confirm that the segmentation algorithm assigns this area to the correct tissue class.

The fourth case is that of a heterogeneous specimen, where multiple tissue types can be visualized by eye. The fluorescent contrast agent showed a small region of potentially positive margins, which warranted more in depth analysis. However, histological annotation showed the presence of only multiple benign breast lobules and not of cancer. OCT also showed some small false positives and the lobules areas. However, RCM confirmed the presence of benign tissue.

Histology correlation of the imaging findings was difficult, as perfect correlation of the OCT/RCM images with histology was almost impossible due to alterations of tissue alignment with processing of the tissue during histology preparation. This is particularly apparent in samples with high content of adipose tissue. For this reason, precise percentages of tissue type area coverage were not computed or compared. Furthermore, the histological practice often outlines large areas that have a mix of tissues within them out of an abundance of caution and to ensure that no tumor cells remain. For example, fat cells within a tumor area, would be segmented as fat cells even though they would be located in a region marked for removal.

The segmentation algorithm correctly classified many complex tissue types as benign, such as fibrosis and treatment-related change from malignant to benign. However, some false positives after OCT image segmentation were still present in two cases. As RCM cannot be performed fast enough on large areas highlighted by FL imaging, this technology needs further improvements to eliminate the false positives. Potentially, larger training sets may improve the false positive rate in areas of normal lobules. More precise histology alignment and preparation will potentially allow for registration of images and tissue type local area comparisons.

Taken together, this case study suggests the novel multimodal instrument can be of great use in identifying surgical margins on breast cancer resection specimens. As noted, rapid feedback of margin extent and location could reduce the need for repeated surgeries and cosmesis damage.

5. Conclusion

Multimodal optical imaging was performed on breast surgical specimens with the goal of evaluating the potential of this technology for assessing surgical margins as a tool for surgery guidance. Fourteen specimens were evaluated and negative findings were not noted. However, overestimation of positive margins was noted on several tissue specimens as compared to histology findings. Although some of the overestimates might indeed be false-positives, it is also possible that they were true-positives, considering perfect histology correlation was impossible due to registration obstacles. Some small false-positives were flagged by OCT algorithmic segmentation in areas of normal lobules. RCM analysis correctly rejected these false positives. Histology processing usually distorts the shape of the specimen, particularly in tissues high in adipocytes. In addition, a single histology slide was taken from each surface of the surgical specimen from a depth of approximately 100 um beneath the specimen surface, while RCM and OCT imaging was performed to depths of 100 μm and 1mm, respectively. The deeper, volumetric information sometimes revealed margins that extended beyond the histology indication. Further evaluation of this technology on additional specimens and better correlation with histology is ongoing which will be reported in a follow-up study. Nevertheless, the preliminary findings are encouraging, showing the potential benefits of this technology for intraoperative evaluation of breast surgical specimens.

6. Limitations and Future Work

Despite encouraging preliminary results, improvements on instrumentation, data collection and data analysis will be considered for future studies. Registration of OCT/RCM images and histological sectioning has to be reconsidered. The specimens here were photographed and marked with a dot of tissue dye to aid in alignment for sectioning. However, there are undoubtedly slight misalignments in pitch and roll rotations. This affects the accuracy of the matching tumor percentages globally and spatially between RCM/OCT and histology. Future studies will attempt to utilize custom cassettes during fixation or imaging and sectioning will be performed using frozen sections within the same laboratory space as soon as possible for better correlation of histology and imaging. The processing algorithm will also needs to be based on larger training sets, as there is significant variation

within the scattering properties of tissue specimens belonging to the same tissue class, which contributes to the false-positive rate in normal lobule areas.

This paper presents only limited case studies; further evaluation of the instrument will be performed using more resection specimens. This will greatly improve the image processing and automated classification techniques. As datasets become larger and more diverse, machine learning techniques can be implemented.

References

1. Azu M, Abrahamse P, Katz SJ, Jagsi R, Morrow M. What is an Adequate Margin for Breast-Conserving Surgery? Surgeon Attitudes and Correlates. Ann Surg Oncol. 2010 Feb;17(2):558–63.
2. Blair SL, Thompson K, Rococco J, Malcarne V, Beitsch PD, Ollila DW. Attaining negative margins in breast-conservation operations: is there a consensus among breast surgeons? J Am Coll Surg. 2009 Nov;209(5):608–13.
3. Morrow M. Breast conservation and negative margins: how much is enough? The Breast. 2009 Oct 1;18:S84–6.
4. Meric F, Mirza NQ, Vlastos G, Buchholz TA, Kuerer HM, Babiera GV, et al. Positive surgical margins and ipsilateral breast tumor recurrence predict disease-specific survival after breast-conserving therapy. Cancer. 2003 Feb 15;97(4):926–33.
5. Swanson GP, Rynearson K, Symmonds R. Significance of margins of excision on breast cancer recurrence. Am J Clin Oncol. 2002 Oct;25(5):438–41.
6. Fleming FJ, Hill ADK, Mc Dermott EW, O'Doherty A, O'Higgins NJ, Quinn CM. Intraoperative margin assessment and re-excision rate in breast conserving surgery. Eur J Surg Oncol J Eur Soc Surg Oncol Br Assoc Surg Oncol. 2004 Apr;30(3):233–7.
7. Waljee JF, Hu ES, Newman LA, Alderman AK. Predictors of Re-excision among Women Undergoing Breast-Conserving Surgery for Cancer. Ann Surg Oncol. 2008 Feb 8;15(5):1297.
8. Méndez JE, Lamorte WW, de las Morenas A, Cerda S, Pistey R, King T, et al. Influence of breast cancer margin assessment method on the rates of positive margins and residual carcinoma. Am J Surg. 2006 Oct 1;192(4):538–40.
9. McLaughlin SA, Ochoa-Frongia LM, Patil SM, Cody HS, Sclafani LM. Influence of frozen-section analysis of sentinel lymph node and lumpectomy margin status on reoperation rates in patients undergoing breast-conservation therapy. J Am Coll Surg. 2008 Jan;206(1):76–82.
10. Menes TS, Tartter PI, Mizrachi H, Smith SR, Estabrook A. Touch preparation or frozen section for intraoperative detection of sentinel lymph node metastases from breast cancer. Ann Surg Oncol. 2003 Dec;10(10):1166–70.
11. Cox CE, Ku NN, Reintgen DS, Greenberg HM, Nicosia SV, Wangensteen S. Touch Preparation Cytology of Breast Lumpectomy Margins With Histologic Correlation. Arch Surg. 1991 Apr 1;126(4):490–3.
12. Jacobs L. Positive margins: the challenge continues for breast surgeons. Ann Surg Oncol. 2008 May;15(5):1271–2.
13. Cendán JC, Coco D, Copeland EM. Accuracy of Intraoperative Frozen-Section Analysis of Breast Cancer Lumpectomy-Bed Margins. J Am Coll Surg. 2005 Aug 1;201(2):194–8.
14. McClatchy DM, Rizzo EJ, Meganck J, Kempner J, Vicory J, Wells WA, et al. Calibration and analysis of a multimodal micro-CT and structured light imaging system for the evaluation of excised breast tissue. Phys Med Biol. 2017 Nov 10;62(23):8983–9000.
15. Qiu S-Q, Dorrius MD, de Jongh SJ, Jansen L, de Vries J, Schröder CP, et al. Micro-computed tomography (micro-CT) for intraoperative surgical margin assessment of breast cancer: A feasibility study in breast conserving surgery. Eur J Surg Oncol J Eur Soc Surg Oncol Br Assoc Surg Oncol. 2018;44(11):1708–13.

16. Tan JE, Orel SG, Schnall MD, Schultz DJ, Solin LJ. Role of magnetic resonance imaging and magnetic resonance imaging--guided surgery in the evaluation of patients with early-stage breast cancer for breast conservation treatment. Am J Clin Oncol. 1999 Aug;22(4):414–8.
17. Moschetta M, Telegrafo M, Introna T, Coi L, Rella L, Ranieri V, et al. Role of specimen US for predicting resection margin status in breast conserving therapy. Il G Chir. 2015 Oct;36(5):201–4.
18. Kalinyak JE, Berg WA, Schilling K, Madsen KS, Narayanan D, Tartar M. Breast cancer detection using high-resolution breast PET compared to whole-body PET or PET/CT. Eur J Nucl Med Mol Imaging. 2014 Feb;41(2):260–75.
19. Narayanan D, Madsen KS, Kalinyak JE, Berg WA. Interpretation of positron emission mammography and MRI by experienced breast imaging radiologists: performance and observer reproducibility. AJR Am J Roentgenol. 2011 Apr;196(4):971–81.
20. Thill M, Röder K, Diedrich K, Dittmer C. Intraoperative assessment of surgical margins during breast conserving surgery of ductal carcinoma in situ by use of radiofrequency spectroscopy. Breast Edinb Scotl. 2011 Dec;20(6):579–80.
21. Troyan SL, Kianzad V, Gibbs-Strauss SL, Gioux S, Matsui A, Oketokoun R, et al. The FLARE intraoperative near-infrared fluorescence imaging system: a first-in-human clinical trial in breast cancer sentinel lymph node mapping. Ann Surg Oncol. 2009 Oct;16(10):2943–52.
22. Kennedy KM, Chin L, McLaughlin RA, Latham B, Saunders CM, Sampson DD, et al. Quantitative micro-elastography: imaging of tissue elasticity using compression optical coherence elastography. Sci Rep. 2015 Oct 27;5:15538.
23. Sharma V, Shivalingaiah S, Peng Y, Euhus D, Gryczynski Z, Liu H. Auto-fluorescence lifetime and light reflectance spectroscopy for breast cancer diagnosis: potential tools for intraoperative margin detection. Biomed Opt Express. 2012 Jul 9;3(8):1825–40.
24. Weyers B, Marsden M, Sun T, Bec J, Bewley AF, Gandour-Edwards RF, et al. Fluorescence lifetime imaging for intraoperative cancer delineation in transoral robotic surgery - Weyers - 2019 - Translational Biophotonics - Wiley Online Library [Internet]. [cited 2020 Aug 14]. Available from: https://onlinelibrary.wiley.com/doi/full/10.1002/tbio.201900017
25. Nguyen FT, Zysk AM, Chaney EJ, Kotynek JG, Oliphant UJ, Bellafiore FJ, et al. Intraoperative Evaluation of Breast Tumor Margins with Optical Coherence Tomography. Cancer Res. 2009 Nov 15;69(22):8790–6.
26. Savastru D, Chang EW, Miclos S, Pitman MB, Patel A, Iftimia N. Detection of breast surgical margins with optical coherence tomography imaging: a concept evaluation study. J Biomed Opt. 2014 May;19(5):056001.
27. Thomas G, Nguyen T-Q, Pence IJ, Caldwell B, O'Connor ME, Giltnane J, et al. Evaluating feasibility of an automated 3-dimensional scanner using Raman spectroscopy for intraoperative breast margin assessment. Sci Rep. 2017 19;7(1):13548.
28. Piras D, Xia W, Steenbergen W, van Leeuwen TonG, Manohar S. Photoacoustic Imaging of the Breast Using the Twente Photoacoustic Mammoscope: Present Status and Future Perspectives. IEEE J Sel Top Quantum Electron. 2010 Jul;16(4):730–9.
29. Maloney BW, McClatchy DM, Pogue BW, Paulsen KD, Wells WA, Barth RJ. Review of methods for intraoperative margin detection for breast conserving surgery. J Biomed Opt. 2018;23(10):1–19.
30. Zhang W, Ling D, Tan J, Zhang J, Li L. Expression of urokinase plasminogen activator and plasminogen activator inhibitor type-1 in ovarian cancer and its clinical significance. Oncol Rep. 2013 Feb 1;29(2):637–45.
